# Examining sociodemographic correlates of opioid use, misuse, and use disorders in the *All of Us* Research Program

**DOI:** 10.1371/journal.pone.0290416

**Published:** 2023-08-18

**Authors:** Hsueh-Han Yeh, Cathryn Peltz-Rauchman, Christine C. Johnson, Pamala A. Pawloski, David Chesla, Stephen C. Waring, Alan B. Stevens, Mara Epstein, Christine Joseph, Lisa R. Miller-Matero, Hongsheng Gui, Amy Tang, Eric Boerwinkle, Mine Cicek, Cheryl R. Clark, Elizabeth Cohn, Kelly Gebo, Roxana Loperena, Kelsey Mayo, Stephen Mockrin, Lucila Ohno-Machado, Sheri Schully, Andrea H. Ramirez, Jun Qian, Brian K. Ahmedani

**Affiliations:** 1 Center for Health Policy and Health Services Research, Henry Ford Health, Detroit, Michigan, United States of America; 2 Department of Public Health Sciences, Henry Ford Health, Detroit, Michigan, United States of America; 3 HealthPartners Institute, Bloomington, Minnesota, United States of America; 4 Office of Research and Education, Spectrum Health, Grand Rapids, Michigan, United States of America; 5 Essentia Health, Essentia Institute of Rural Health, Duluth, Minnesota, United States of America; 6 Center for Applied Health Research, Baylor Scott & White Health, Temple, Texas, United States of America; 7 Department of Medicine, University of Massachusetts Medical School, Worcester, Massachusetts, United States of America; 8 Behavioral Health Services, Henry Ford Health, Detroit, Michigan, United States of America; 9 School of Public Health, The University of Texas Health Science Center at Houston, Houston, Texas, United States of America; 10 Department of Laboratory Medicine and Pathology, Mayo Clinic, Rochester, Minnesota, United States of America; 11 Department of Medicine, Brigham and Women’s Hospital, Boston, Massachusetts, United States of America; 12 Hunter-Bellevue School of Nursing, Hunter College, City University of New York, New York, New York, United States of America; 13 Johns Hopkins University School of Medicine, Baltimore, Maryland, United States of America; 14 Vanderbilt Institute for Clinical and Translational Research, Vanderbilt University Medical Center, Nashville, Tennessee, United States of America; 15 All of Us Research Program, National Institutes of Health, Bethesda, Maryland, United States of America; 16 Department of Biomedical Informatics, UCSD Health, La Jolla, California, United States of America; 17 Department of Medicine, Vanderbilt University Medical Center, Nashville, Tennessee, United States of America; 18 Biomedical Informatics, Vanderbilt University Medical Center, Nashville, Tennessee, United States of America; University of New Mexico Health Sciences Center, UNITED STATES

## Abstract

**Background:**

The *All of Us* Research Program enrolls diverse US participants which provide a unique opportunity to better understand the problem of opioid use. This study aims to estimate the prevalence of opioid use and its association with sociodemographic characteristics from survey data and electronic health record (EHR).

**Methods:**

A total of 214,206 participants were included in this study who competed survey modules and shared EHR data. Adjusted logistic regressions were used to explore the associations between sociodemographic characteristics and opioid use.

**Results:**

The lifetime prevalence of street opioids was 4%, and the nonmedical use of prescription opioids was 9%. Men had higher odds of lifetime opioid use (aOR: 1.4 to 3.1) but reduced odds of current nonmedical use of prescription opioids (aOR: 0.6). Participants from other racial and ethnic groups were at reduced odds of lifetime use (aOR: 0.2 to 0.9) but increased odds of current use (aOR: 1.9 to 9.9) compared with non-Hispanic White participants. Foreign-born participants were at reduced risks of opioid use and diagnosed with opioid use disorders (OUD) compared with US-born participants (aOR: 0.36 to 0.67). Men, Younger, White, and US-born participants are more likely to have OUD.

**Conclusions:**

*All of Us* research data can be used as an indicator of national trends for monitoring the prevalence of receiving prescription opioids, diagnosis of OUD, and non-medical use of opioids in the US. The program employs a longitudinal design for routinely collecting health-related data including EHR data, that will contribute to the literature by providing important clinical information related to opioids over time. Additionally, this data will enhance the estimates of the prevalence of OUD among diverse populations, including groups that are underrepresented in the national survey data.

## Introduction

Over the past two decades, opioid use has grown into a national crisis in the United States. Prescription opioid use and illegal street opioid use have increased substantially and have become some of the most concerning national public health problems. The increased use has been seen with both illegal opiate drugs and prescribed opioids [1, 2]. More than 106,000 people died by drug overdose in the United States in 2021; 70% of which were opioid-related, including both prescription and illegal opioids [3]. Monitoring the prevalence of opioid use becomes crucially important for understanding the trend of use over time, for planning the needs of opioid use disorders (OUD) treatment, implementing prevention initiatives, and evaluating the effectiveness of addiction treatment programs. However, the estimated prevalence of opioid use tends to vary across different studies and data sources [2, 4–6]. For example, National Survey on Drug Use and Health (NSDUH) estimates in 2012 and 2013 were higher than the National Epidemiologic Survey on Alcohol and Related Conditions (NESARC) estimates from 2012 to 2013 for lifetime heroin use (2% vs. 1.6%). Past-year heroin use was 1.5 times higher from NSDUH as compared to estimates from NESARC data (0.3% vs. 0.2%) [2, 4, 7]. The NSDUH estimates for past-year non-medical prescription opioid use during 2012–2013 ranged from 4.9% to 5.6% [5], and these estimates were higher than the estimates of 4% from NESARC-III in 2012–2013 [6]. To have a better understanding of the opioid problem from the numbers of affected individuals, more studies from diverse populations and data sources are needed.

The *All of Us* Research Program, launched by the National Institutes of Health in 2018, aims to enroll one million or more people living in the United States to gather one of the richest health data in history to accelerate research that may help improve health. This also provides a unique opportunity to conduct studies of opioid use and OUD given focus on engagement of populations from underrepresented groups including racial and ethnic backgrounds [8].

This study explored the opioid use in the *All of Us* Research Program, using both self-reported survey data and EHR data. Diagnosis and prescription data were collected through the EHR systems of affiliated health systems. The participants also provided information on the Lifestyle Survey Module, administered at baseline enrollment, including lifetime use and past 3-month use for both prescription opioids and street opioids [9]. We also investigate the sociodemographic disparities in opioid use to better understand each group within the *All of Us* cohort.

## Materials and methods

The goals, recruitment methods and sites, and scientific rationale for *All of Us* Research Program, and the details of recruitment and enrollment were published previously [8]. The program was not designed with a formal statistical sampling method, and the participants are not recruited through probability-based sampling. Therefore, it cannot be considered as a representative sample. Instead, the program invites a diverse range of participants through various avenues, including members of groups that have been underrepresented in biomedical research in the past. Participants are recruited voluntarily through a wide range of outreach approaches, including through affiliated healthcare provider organizations and community health centers nationwide. Additionally, interested individuals have the option to directly enroll through the *All of Us* website (JoinAllofUs.org) or by attending *All of Us* events. After enrollment forms are completed online (https://joinallofus.org), participants are given informed electronic consent modules including explanatory videos with brief text and formative questions. Participants are then asked to complete several surveys including the Lifestyle Survey Module about their lifetime and recent substance use behaviors [9]. Participants also consent to share biospecimen samples and EHR data with the program for research purposes during the enrollment. This study is one of the *All of Us* demonstration projects. The demonstration projects were designed to describe the cohort, replicate previous findings from national studies for validation, and avoid novel discovery, in line with the program values to ensure equal access by researchers to the data. The work described here was proposed by Consortium members, reviewed, and overseen by the program’s Science Committee. The initial release of data and tools used in this work was published recently. Results reported are in accordance with the policy of *All of Us* Data and Statistics Dissemination with prohibition against disclosing the number of group counts under 20. The *All of Us* IRB had designated available data used in this study as meeting criteria for non-human subject research.

This study used information from the lifestyle survey questionnaire and EHR drug exposure table to estimate opioid use in the *All of Us*. Approximately 50% of the participants allowed access to their EHR data. The lifestyle survey asked questions about participants’ lifetime and current use of tobacco, alcohol, and other drugs including street opioids and nonmedical use of prescription opioids. For example, the question series starts with “In your lifetime, have you ever used street opioids (e.g., heroin, opium)?” The response was either “yes” or “no.” For those participants who have used street opioids in their lifetime, the additional question was asked: “In the past three months, how often have you used it?” The answer was categorized into “never”, “once or twice”, “monthly”, “weekly”, or “daily or almost daily” [9]. The Observational Medical Outcomes Partnership Common Data Model (OMOP CDM) is used in the *All of Us* program to ensure all EHR data is standardized for analysis across sites [10, 11]. Participant’s privacy is protected through the entire data process. The EHR drug exposure table captures records of medications including prescription, over-the-counter medicines, vaccines, and large-molecule biologic therapies. The information in this table captures medication orders from electronic ordering systems, and prescriptions filled at pharmacies via reimbursement from claims systems. Conditions of OUD were defined according to the Systematized Nomenclature of Medicine—Clinical Terms (SNOMED-CT) [12]. Prescription opioids in this study include Butorphanol, Codeine, Fentanyl, Hydrocodone, Hydromorphone, Meperidine, Morphine, Nalbuphine, Oxycodone, Propoxyphene, and Tramadol. OUD diagnoses were selected from all relevant ICD-10-CM codes from the EHR condition table.

Eligible participants in this study were those who shared their EHR and answered the Lifestyle Survey Module. A total of 214,206 participants aged 18 years or older who were recruited by the end of 2019 were included in the analysis. In the Basic Survey Module, participants were asked to identify themselves as Asian, Black or African American, Hispanic or Latino, White, or Others. The birth country and state of current residence were also reported. Prevalence is presented as a proportion with 95% binomial confidence intervals. Logistic regression was used to explore sociodemographic disparities. The significance level was defined as p-value < 0.05 (two-tailed).

The *All of Us* Research Workbench platform (https://www.researchallofus.org/data-tools/workbench), used for the analysis in this study, provides researchers with an environment for running Jupyter notebooks. Our research team members have been authorized as registered users by the *All of Us* research program and can access the Registered Tier data and tools. This work was approved and reviewed by the *All of Us* Research Program Science Committee and the Data and Research Center to ensure compliance with program policy. The t-tests and chi-square analyses were conducted to examine the descriptive differences in demographic characteristics, and logistic regression was used to examine the relationship between demographic characteristics and opioid use, misuse, and use disorders. All analyses were performed by R in Jupyter notebooks.

## Results

Table 1 summarize the sociodemographic characteristics and opioid use among the eligible study sample (n = 214,206). According to the self-reported survey, 4% have used street opioids (e.g., heroin, opium) in their lifetime; 0.07% reported past 3 months use. Approximately 9% reported nonmedical use of prescription opioids (NMUPO) in their lifetime; 0.24% reported past 3 months use. Approximately 14% received prescription opioid and 1% had diagnoses of OUD in the past 3 months from the providers.

**Table 1:** Sociodemographic characteristics of the *All of Us* Research Program among participants who completed lifestyle survey and shared their EHR data. Prevalence (%) and 95% Confidence Interval (95% CI) of opioids use and OUD in the *All of Us* Research Program among groups of gender, age, race/ethnicity, country of origin, and region of residence.

		Survey Data ^b^				Electronic Health Records (EHR) ^b^	
	All of Us Survey-EHR Cohort ^a^	Street Opioids Use		Nonmedical Use of Rx Opioids		Rx Opioid received in the past 3 months	OUD Diagnosis in the past 3 months
		Lifetime	Past 3 months	Lifetime	Past 3 months		
	**n = 214,206**	**n = 9,504**	**n = 145**	**n = 18,699**	**n = 505**	**n = 29,659**	**n = 2,602**
	n (%)	% (95% CI)	% (95% CI)	% (95% CI)	% (95% CI)	% (95% CI)	% (95% CI)
**Gender**							
Women	130,538 (60.94)	2.61 (2.53, 2.70)	0.04 (0.03, 0.05)	7.91 (7.76, 8.06)	0.27 (0.25, 0.30)	13.85 (13.66, 14.04)	0.87 (0.82, 0.92)
Men	79,548 (37.14)	7.31 (7.13, 7.50)	0.11 (0.09, 0.14)	10.05 (9.84, 10.26)	0.17 (0.14, 0.20)	13.82 (13.58, 14.06)	1.73 (1.64, 1.83)
**Age (Years)**							
18–39	59,115 (27.60)	5.50 (5.32, 5.69)	---	10.77 (10.52, 11.02)	0.13 (0.10, 0.16)	12.02 (11.76, 12.28)	1.51 (1.41, 1.61)
40–49	31,385 (14.65)	5.83 (5.57, 6.09)	0.10 (0.07, 0.15)	11.25 (10.91, 11.61)	0.30 (0.25, 0.37)	13.81 (13.43, 14.20)	1.81 (1.67, 1.96)
50–59	44,313 (20.69)	4.89 (4.69, 5.10)	0.14 (0.11, 0.18)	8.94 (8.68, 9.21)	0.38 (0.32, 0.44)	15.13 (14.79, 15.46)	1.46 (1.35, 1.57)
60–69	45,469 (21.23)	4.08 (3.90, 4.26)	0.08 (0.06, 0.11)	7.33 (7.09, 7.57)	0.22 (0.18, 0.27)	15.06 (14.73, 15.39)	0.92 (0.84, 1.02)
≥ 70	33,924 (15.84)	1.19 (1.08, 1.31)	---	4.44 (4.23, 4.67)	0.20 (0.16, 0.26)	13.77 (13.40, 14.14)	0.23 (0.18, 0.29)
**Race/Ethnicity**							
White	110,152 (51.42)	4.81 (4.69, 4.94)	---	10.97 (10.78, 11.15)	0.18 (0.16, 0.21)	14.81 (14.60, 15.02)	1.31 (1.24, 1.38)
Black or African American	45,151 (21.08)	4.62 (4.43, 4.82)	0.21 (0.18, 0.26)	6.03 (5.81, 6.25)	0.34 (0.29, 0.40)	12.26 (11.96, 12.57)	1.04 (0.95, 1.14)
Hispanic or Latino	42,014 (19.61)	3.53 (3.36, 3.71)	0.06 (0.04, 0.09)	6.27 (6.04, 6.50)	0.26 (0.22, 0.32)	13.72 (13.39, 14.06)	1.18 (1.08, 1.28)
Asian	5,944 (2.77)	0.82 (0.62, 1.10)	---	4.07 (3.59, 4.61)	---	8.06 (7.39, 8.79)	---
**Country of origin**							
US born	178,534 (83.35)	5.03 (4.93, 5.13)	0.08 (0.06, 0.09)	9.79 (9.66, 9.93)	0.24 (0.22, 0.26)	14.44 (14.28, 14.61)	1.36 (1.3, 1.41)
Not US born	33,460 (15.62)	1.14 (1.03, 1.26)	---	2.99 (2.82, 3.18)	0.21 (0.16, 0.26)	10.64 (10.31, 10.97)	0.36 (0.30, 0.44)
**Region of residence** ^c^							
West	56,326 (26.30)	5.17 (4.99, 5.35)	---	11.01 (10.75, 11.27)	0.30 (0.25, 0.35)	21.17 (20.83, 21.51)	1.12 (1.03, 1.21)
South	39,409 (18.40)	3.08 (2.91, 3.25)	---	7.44 (7.18, 7.70)	0.26 (0.22, 0.32)	7.89 (7.63, 8.16)	0.51 (0.44, 0.59)
Midwest	43,146 (20.14)	4.26 (4.07, 4.45)	0.20 (0.16, 0.25)	7.33 (7.09, 7.58)	0.17 (0.14, 0.22)	11.92 (11.62, 12.23)	0.74 (0.66, 0.82)
Northeast	65,264 (30.47)	4.92 (4.76, 5.09)	0.05 (0.04, 0.08)	8.69 (8.47, 8.91)	0.22 (0.19, 0.26)	12.69 (12.43, 12.94)	2.09 (1.98, 2.20)

^a^ Percentages may not sum to 100 because of missing data

^b^ The prevalence with the number of patients <20 was not shown in this table according to the policy of All of Us Data and Statistics Dissemination

^c^ West: Alaska, Arizona, California, Colorado, Hawaii, Idaho, Montana, Nevada, New Mexico, Oregon, Utah, Washington, Wyoming; South: Alabama, Arkansas, Delaware, Florida, Georgia, Kentucky, Louisiana, Maryland, Mississippi, North Carolina, Oklahoma, South Carolina, Tennessee, Texas, Virginia, West Virginia, Washington, D.C.; Midwest: Illinois, Indiana, Iowa, Kansas, Michigan, Minnesota, Missouri, Nebraska, North Dakota, Ohio, South Dakota, Wisconsin; Northeast: Connecticut, Maine, Massachusetts, New Hampshire, New Jersey, New York, Pennsylvania, Rhode Island, Vermont

Table 1 also presents the prevalence of opioid use and OUD diagnosis by sociodemographic groups. Prevalence of lifetime and current street opioid use were higher among men (7.3%; 95% CI: 7.13%, 7.50%) than women (2.6%; 95% CI: 2.53%, 2.70%). Men also had higher rate of lifetime NMUPO. However, the rate of NMUPO in the past 3 months is higher among women (0.27%; 95% CI: 0.25%, 0.30%) than men (0.17%; 95% CI: 0.14%, 0.20%). 1.7% of men received OUD diagnosis in the past 3 months as compared to 0.9% for women. Higher rates of opioid use or OUD were found among young adults compared to elderly population. However, rates of prescription opioids from providers were higher among the older group. Prevalence of lifetime street opioid use were higher among Non-Hispanic Whites and Non-Hispanic Blacks (4%~5%). Non-Hispanic Whites also had the highest rates of lifetime NMUPO (11%) and OUD (1.31%). US-born participants had higher rates of lifetime street opioid use (5%) and NMUPO (9.8%), and higher rates of receiving prescription opioids (14.4%) and OUD (1.36%). Participants in the West or Northeast regions had higher rates of lifetime street opioid use (4~5%) and NMUPO (9~11%). Those who lived in the West had highest rate of receiving prescription opioids from the providers (21.2%), and those in the Northeast had the highest rate of OUD (2.1%).

We further investigated the association of sociodemographic disparities, with adjusting for sociodemographic factors in the model. The adjusted odds ratio (aOR) was presented in the Table 2 as measure of the association for each outcome. Men had higher odds of lifetime street opioid use (aOR: 3.11; 95% CI: 2.98, 3.25), lifetime NMUPO (aOR: 1.36; 95% CI: 1.32, 1.40), and receiving OUD diagnosis (aOR: 2.13; 95% CI: 1.96, 2.30), but less likely to have past-3-months NMUPO (aOR: 0.58; 95% CI: 0.47, 0.71). Participants from other racial and ethnic categories were at reduced odds of lifetime opioid use or OUD compared with non-Hispanic White participants (aOR: 0.3 to 0.86). However, the non-Hispanic Black or Hispanic participants were more likely to have current use of street opioid or NMUPO (aOR: 1.9 to 9.9). Foreign-born participants were at reduced risk for opioid use, receiving prescription opioids, and OUD diagnoses compared with participants who were born in the US (aOR: 0.36 to 0.67).

**Table 2:** Adjusted odds ratio (aOR) and 95% confidence interval (95% CI) for opioids use and OUD.

	Survey Data				Electronic Health Records (EHR)	
	Street Opioids Use		Nonmedical Use of Prescription Opioids		Rx Opioid received in the past 3 months aOR (95% CI)	OUD Diagnosis in the past 3 months aOR (95% CI)
	Lifetime aOR (95% CI)	Past 3 months aOR (95% CI)	Lifetime aOR (95% CI)	Past 3 months aOR (95% CI)		
**Gender**						
Women	Ref.	Ref.	Ref.	Ref.	Ref.	Ref.
Men	3.11 (2.98, 3.25)	2.78 (1.97, 3.96)	1.36 (1.32, 1.40)	0.58 (0.47, 0.71)	0.98 (0.95, 1.00)	2.13 (1.96, 2.30)
**Age (Years)**						
18–39	Ref.	Ref.	Ref.	Ref.	Ref.	Ref.
40–49	1.07 (1.01, 1.14)	3.74 (2.06, 7.13)	1.09 (1.04, 1.14)	2.50 (1.84, 3.39)	1.21 (1.16, 1.26)	1.25 (1.12, 1.39)
50–59	0.83 (0.78, 0.87)	4.53 (2.63, 8.32)	0.81 (0.78, 0.85)	3.18 (2.42, 4.22)	1.35 (1.31, 1.40)	0.97 (0.87, 1.07)
60–69	0.63 (0.59, 0.67)	3.23 (1.79, 6.12)	0.58 (0.55, 0.61)	2.01 (1.49, 2.74)	1.32 (1.27, 1.37)	0.57 (0.50, 0.64)
≥ 70	0.16 (0.14, 0.17)	---	0.29 (0.28, 0.31)	2.08 (1.48, 2.92)	1.15 (1.10, 1.20)	0.12 (0.10, 0.16)
**Race/Ethnicity**						
White	Ref.	Ref.	Ref.	Ref.	Ref.	Ref.
Black or African American	0.76 (0.72, 0.81)	9.86 (6.10, 16.88)	0.44 (0.42, 0.46)	1.83 (1.48, 2.27)	0.80 (0.77, 0.82)	0.62 (0.55, 0.69)
Hispanic or Latino	0.89 (0.83, 0.94)	4.89 (2.56, 9.44)	0.63 (0.60, 0.66)	1.88 (1.43, 2.45)	1.13 (1.08, 1.17)	1.04 (0.93, 1.17)
Asian	0.24 (0.18, 0.31)	---	0.48 (0.42, 0.54)	1.57 (0.78, 2.82)	0.68 (0.61, 0.75)	0.20 (0.10, 0.35)
**Country of origin**						
US born	Ref.	Ref.	Ref.	Ref.	Ref.	Ref.
Not US born	0.25 (0.22, 0.27)	0.36 (0.16, 0.72)	0.33 (0.30, 0.35)	0.63 (0.46, 0.85)	0.66 (0.63, 0.68)	0.26 (0.21, 0.31)

aOR and 95% CI in grey color indicates the non-significant association with outcomes

## Discussion

The *All of Us* provides a novel research resource that includes EHR data and survey responses. The program aims to recruit diverse participants that include members of groups that have been underrepresented in biomedical research in the past such as racial and ethnic minority groups, elderly groups, and those who were foreign-born. We investigated opioid use in the *All of Us* to explore who is affected, examining prevalence and the association of sociodemographic disparities with various definitions of opioid use.

The prevalence in the *All of Us* vary slightly compared to other data sources [2, 5, 13–15]. NSDUH and NESARC have reported 1~2% of individuals used heroin at least once in their lifetime [2, 13]. *All of Us* includes all street opioids (i.e., heroin and other opium), yielding the lifetime prevalence of 4%. However, the prevalence of current opioid use is lower in *All of Us* compared with the prevalence from NSDUH [13]. The most current NESARC data was collected in 2012–2013, which might not reflect the rise in opioids nationally in the past decade. This difference might also be due to the diversity in the study population [13, 15, 16]. Participants enrolled in the *All of Us* program may differ from national survey samples due to study inclusion criteria, as most surveys leave out some parts of the population. For example, NSDUH survey does not include homeless individuals who do not utilize shelters, active-duty military personnel, and individuals residing in institutional group quarters such as prisons and hospitals. On the other hand, *All of Us* recruits anyone who is 18 years or older and are currently living in the United States. Given that national surveys exclude certain marginalized populations who may be at higher risks of opioid use, *All of Us* has the potential to address this gap by recruiting a more diverse population from a combination of both the community and the healthcare systems. This inclusive approach enables the program to encompass individuals who were previously overlooked, leading to a more comprehensive understanding of opioid-related issues [17].

In response to the opioid epidemic, *All of Us* Research Data can also be used as an indicator of national trends for monitoring the prevalence of receiving prescription opioids and nonmedical use of opioids in the United States. *All of Us* employs a longitudinal design for collecting health-related data from all eligible participants, therefore researchers will be able to investigate and evaluate the addition of genetic and environmental factors on the development of disease as well as precision medicine approaches to prevention and treatment of substance use disorders for the future research [18].

It is important to note that the study samples included in the analyses are those who are enrolled in *All of Us*, have consented to share their EHR data for research purposes, and completed the *All of Us* lifestyle survey questionnaire. Selection bias may impact the results, although our findings remain consistent with other studies. The prevalence was estimated from the data captured in the *All of Us* research data workbench. Some participants may not recall or not report their data on surveys, consistent with other large survey-based studies. In addition, *All of Us* Research Program includes a demographically, geographically, and medically diverse group of participants; however, it is not a representative sample of the population of the United States. Despite these limitations, this study demonstrates the potential value of the cohort for future large-scale research. *All of Us* Research Data can also be used as an indicator of national trends for monitoring the prevalence of receiving prescription opioids and non-medical use of opioids in the United States. Furthermore, researchers will be able to investigate and evaluate the addition of genetic and environmental factors on the development of disease as well as precision medicine approaches to prevention and treatment of substance use disorders in the future because the *All of Us* employs a longitudinal design for collecting health-related data from all eligible participants [18].
